# Evaluating the prevalence of eating disorder risk and low energy availability risk in collegiate athletes

**DOI:** 10.1186/s40337-025-01218-w

**Published:** 2025-03-21

**Authors:** Lauren J. Puscheck, Julie Kennel, Catherine Saenz

**Affiliations:** 1 Department of Human Sciences, Human Nutrition Program, The Ohio State University, 306B Atwell Hall, 453 W 10th Ave, Columbus, OH 43210 USA; 2 Delaware Athletics, Community and Campus Recreation, University of Delaware, 631 S College Ave, Newark, DE 19716 USA; 3 Department of Human Sciences, Exercise Science Program, The Ohio State University, A048 PAES Building, 305 Annie and John Glenn Ave, Columbus, OH 43210 USA

**Keywords:** Eating disorders, Low energy availability, LEA, Relative energy deficiency in sport, REDs, Collegiate athletes

## Abstract

**Background:**

Eating disorders and low energy availability independently and negatively impact eating behaviors and attitudes and overall health. Screening provides early identification of athletes suffering from these conditions, yet few studies have evaluated both simultaneously, and none have investigated eating disorder and low energy availability in the context of sex or sport type. This study determined the prevalence of eating disorder and low energy availability risk in collegiate athletes at a large National Collegiate Athletic Association (NCAA) Division 1 university and the variation of prevalence by sport, sport type, and sex.

**Methods:**

This cross-sectional study of NCAA Division 1 athletes used a self-administered survey to collect demographic data and assessed risk using the Eating Disorder Screen for Athletes, Low Energy Availability in Females Questionnaire, and Low Energy Availability in Males Questionnaire. Variations in prevalence were assessed using chi-square tests and variations in mean screening tool scores were assessed using t-tests and ANOVA. Significance was set at *p* < 0.05.

**Results:**

Out of 220 athletes (76 males, 144 females) across 19 sports, 38.18% were at risk for an eating disorder and 47.17% were at risk for low energy availability, while 22.73% were at risk for both conditions. Females had a higher distribution of positive screens for an eating disorder (*p* = 0.002) and low energy availability (*p* < 0.001) than males. Aesthetic sport athletes had the highest proportion of eating disorder risk (*p* = 0.016)—but low energy availability (*p* = 0.871) did not vary by sport type. Dance team athletes had the highest prevalence of eating disorder risk by sport (*p* < 0.001), while women’s gymnasts had the highest prevalence of low energy availability risk (*p* < 0.001) by sport.

**Conclusions:**

A high percentage of athletes were at risk for an eating disorder and low energy availability. Screening protocols can identify eating disorders and low energy availability earlier, leading to quicker treatment and prevention of severe consequences.

## Abstract (plain language summary)

Eating disorders (ED) are medical illnesses that cause severe disturbances to one’s eating patterns and behaviors, affecting physical, mental, and social health. Athletes face sport-specific pressures on top of general risk factors that may lead to eating disorders. Another concern for athletes is low energy availability (LEA), which—when prolonged—can lead to the development of Relative Energy Deficiency in Sport (REDs). Both conditions can lead to serious health and performance consequences. Screening for eating disorders and low energy availability is important for early identification and treatment to prevent the severe consequences of these conditions. The aims of the study were to determine the prevalence of ED and LEA risk in collegiate athletes at an NCAA Division 1 university and to analyze how risk differs by sport, sport type, and sex. The results indicate that male and female collegiate athletes across a variety of sports and sport types are at risk for ED and LEA. Females had a higher risk prevalence for both ED and LEA. By sport type, aesthetic athletes had the highest prevalence for ED risk, while by sport, dance team athletes had the highest prevalence of ED risk and women’s gymnasts had the highest prevalence of LEA risk.

## Introduction

Eating disorders (ED) are medical illnesses that cause severe disturbances to one’s eating patterns and behaviors; affect one’s physical, mental, and social health; and lead to serious health consequences including osteopenia and osteoporosis, gastrointestinal distress, electrolyte imbalances, muscle wasting, chronic fatigue, and cardiac abnormalities [1–3]. Athletes with an eating disorder can face decreases in athletic performance on top of the general health consequences of these illnesses [3, 4].

Prolonged low energy availability (LEA) describes a period when energy intake does not meet an athlete’s total energy needs, particularly due to high exercise energy expenditure, leading to impaired physiological functioning that could eventually develop into Relative Energy Deficiency in Sport (REDs) [5]. REDs impairs numerous body functions including energy metabolism, musculoskeletal recovery, immunity, injury prevention and recovery, reproductive function, glycogen synthesis, and many others, which all hinder an athlete’s physical performance and overall health [5].

Therefore, both ED and LEA lead to acute and potentially chronic health detriments, decreased recovery processes, and increased injury risks [3, 5–9]. The additional sport-specific pressures that collegiate athletes face result in increased risk for developing either or both of these conditions, especially when combined with high training demands and academic stressors [4, 5, 10–13]. This may further exacerbate the negative impacts on a collegiate athlete’s academic progress, cognitive well-being, sleep, and overall recovery [3, 5, 9]. Therefore, addressing ED and LEA is paramount to athlete wellness and development.

Prior research has concluded that there is no significant difference in disordered eating prevalence between athletes and non-athletes [14, 15]. However, male elite athletes in weight class, anti-gravitation, lightweight, and combat and contact sports may have a higher rate of disordered eating than male athletes in other sport types [14]. Female athletes in aesthetic and lean sports may have higher rates of eating disorder psychopathology and symptoms than those in non-aesthetic and non-lean sports [15]. Female athletes tend to have a higher prevalence of subclinical and clinical eating disorders than male athletes [4, 16–19]. Studies evaluating the prevalence of LEA and REDs are limited as this is a growing area of research, especially in male athletes, but the existing studies that have evaluated the prevalence of LEA have reported rates over 50% in female athletes and 32% in male athletes [5, 20–23]. Limited research exists related to the prevalence of LEA or REDs risk by sport and sport type, although prevalence rates for LEA above 50% have been reported in endurance sports, softball, ballet, beach volleyball, indoor volleyball, and equestrian athletes [20, 22].

The unique setting of collegiate athletics presents a wide range of sports and athletes, highlighting the need for streamlined processes to target and address ED and LEA risk. Identifying at-risk athletes through accessible screening options, such as self-reported surveys, could deter the development and/or severity of these often-concurrent conditions [24, 25]. Additionally, studying the prevalence of ED and LEA in the collegiate setting could aid athletic departments in improving their support of student athletes’ health, well-being, and performance. While several surveys exist for this purpose, limited research is available for how these conditions present in collegiate athletic settings and even less is available to identify how these conditions present in subgroups related to sex, sport, or sport type. The aim of the present study was to determine the prevalence rates of ED risk and LEA risk in collegiate athletes at a large Division 1 university in the Midwest. The prevalence rates were analyzed by sport, sport type, and sex to evaluate how risk differs amongst these variables and which athletes are at the highest risk for ED and LEA.

## Methods

### Study design and participants

**Fig. 1:**
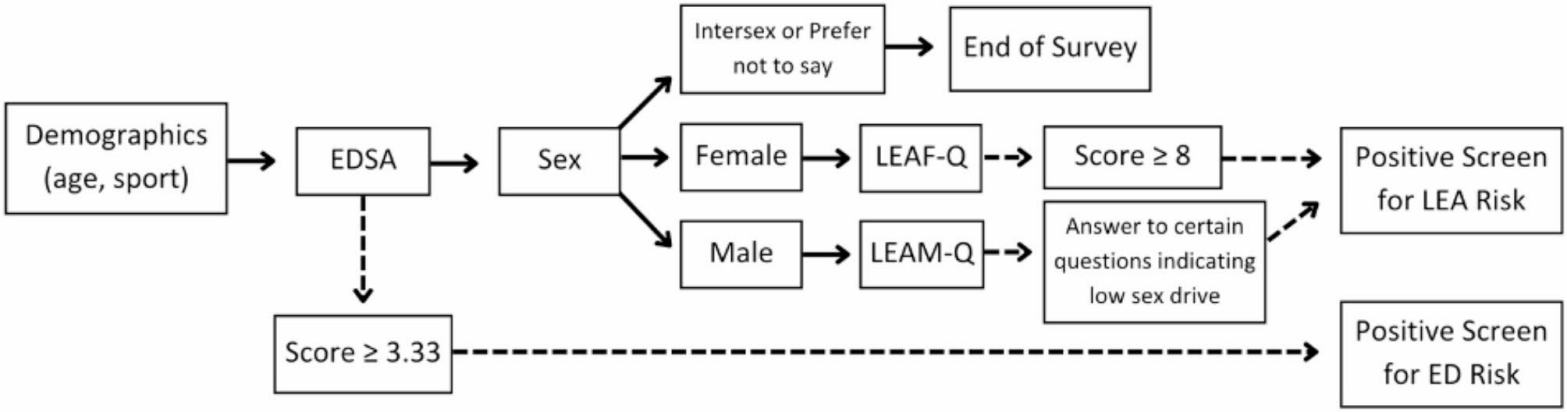
Survey flow. Data collection survey order and logic. EDSA, Eating Disorder Screen for Athletes [16]; LEAM-Q, Low Energy Availability in Males Questionnaire [23]; LEAF-Q, Low Energy Availability in Females Questionnaire [22]

This cross-sectional study was a multi-part survey delivered via Qualtrics 2023. Inclusion criteria for the study were Division 1 athletes at the university over 18 at the time of data collection. Digital informed consent was obtained. The survey included basic demographic questions (age, sport played at the university, sex assigned at birth, and gender), the Eating Disorder Screen for Athletes (EDSA) to screen for eating disorder risk, and the Low Energy Availability in Females Questionnaire (LEAF-Q) or Low Energy Availability in Males Questionnaire (LEAM-Q) to screen for low energy availability risk (Fig. 1). The survey did not collect names to deter false answers and under-reporting. The study was considered exempt by the university’s Institutional Review Board. To recruit participants, each team was presented with a study overview and then provided a QR code with the survey link.

The sport types identified and evaluated in this study were aesthetic, endurance, and ball. Aesthetic and endurance sports tend to emphasize leanness for enhanced sport performance. Ball sports are generally viewed as non-lean sports. Table 1 presents the categorization of sports by sport type. Technical sports are another existing sport category generally viewed as non-lean; however, the response rate of athletes in technical sports was too low to include in analysis.

**Table 1:** Sport type composition and sample size

Sport Type	Sports Included	Sample Size
Aesthetic	Dance, women’s gymnastics, men’s gymnastics, synchronized swimming	*n* = 39 2 males, 37 females
Endurance	Rowing, swimming, track events	*n* = 25 8 males, 17 females
Ball	women’s ice hockey, men’s ice hockey, women’s soccer, field hockey, softball, men’s volleyball, women’s lacrosse, women’s volleyball, women’s basketball	*n* = 112 28 males, 84 females

The sport types evaluated for data analysis and their individual sport composition and sample size

### Data collection

Any sport with at least 10 responses for both EDSA and LEA screening was identified and evaluated for prevalence rates by specific sport. Any sports that did not have at least 10 responses were grouped together as “other sports”. The other sports data was not subjected to statistical testing as the group consisted of heterogenous sports and differed in number of responses from each included sport. Swimming was presented as a specific sport, but it was not included in analysis since there were less than 10 responses for each sex in the sample.

Raw survey answers were converted to scores based on the validated scoring procedure and score key for each screening tool [16, 22, 23]. The EDSA uses an average score of 6 questions from a 1–5 Likert scale [16]. Scores on the EDSA range from 1.00 to 5.00, with a score of 3.33 or higher for a positive screen. LEAF-Q uses a total score summed from each of the scored questions [22]. LEAF-Q scores range from 0 to 37, with a cut-off score of 8 or higher for a positive screen. The only subsection on the LEAM-Q that is validated with adequate sensitivity and specificity for LEA risk is the sex drive subsection [23]. To screen positive for LEA, male athletes must indicate a low sex drive or rare morning erections of which the frequency is less than usual.

The major outcomes evaluated were average EDSA, LEAF-Q, and LEAM-Q sex drive scores; proportion of positive EDSA, LEAF-Q, and LEAM-Q screens; and proportion of positive screens for both ED and LEA risk within the same individual.

### Statistical analysis

After data was collected and EDSA and LEA scores were calculated, statistical analysis was conducted in Microsoft Excel and IBM SPSS Version 26 to determine significant differences between groups in the data. Statistical differences in the proportion of positive screens for both ED risk and LEA risk by sex, sport type, and specific sport were evaluated by chi-squared testing. Mean EDSA scores were analyzed for differences in sex using a two-tailed t-test and for differences in sport type and sport using one-way ANOVA with Tukey’s post-hoc. One-way ANOVA with Tukey post-hoc testing was also used to determine differences of LEA average scores by sport for the female sports as five female sports were compared. A two-tailed t-test was used to determine differences of LEA average scores by specific sport for the male sports as only two male sports were compared. Variances across groups were assessed with a test of homogeneity of variances, and the significance of the ANOVA testing for EDSA average scores and LEA average scores by sport for the female sports were confirmed with a Welch’s test. Significance for all tests was set at *p* < 0.05.

## Results

**Fig. 2:**
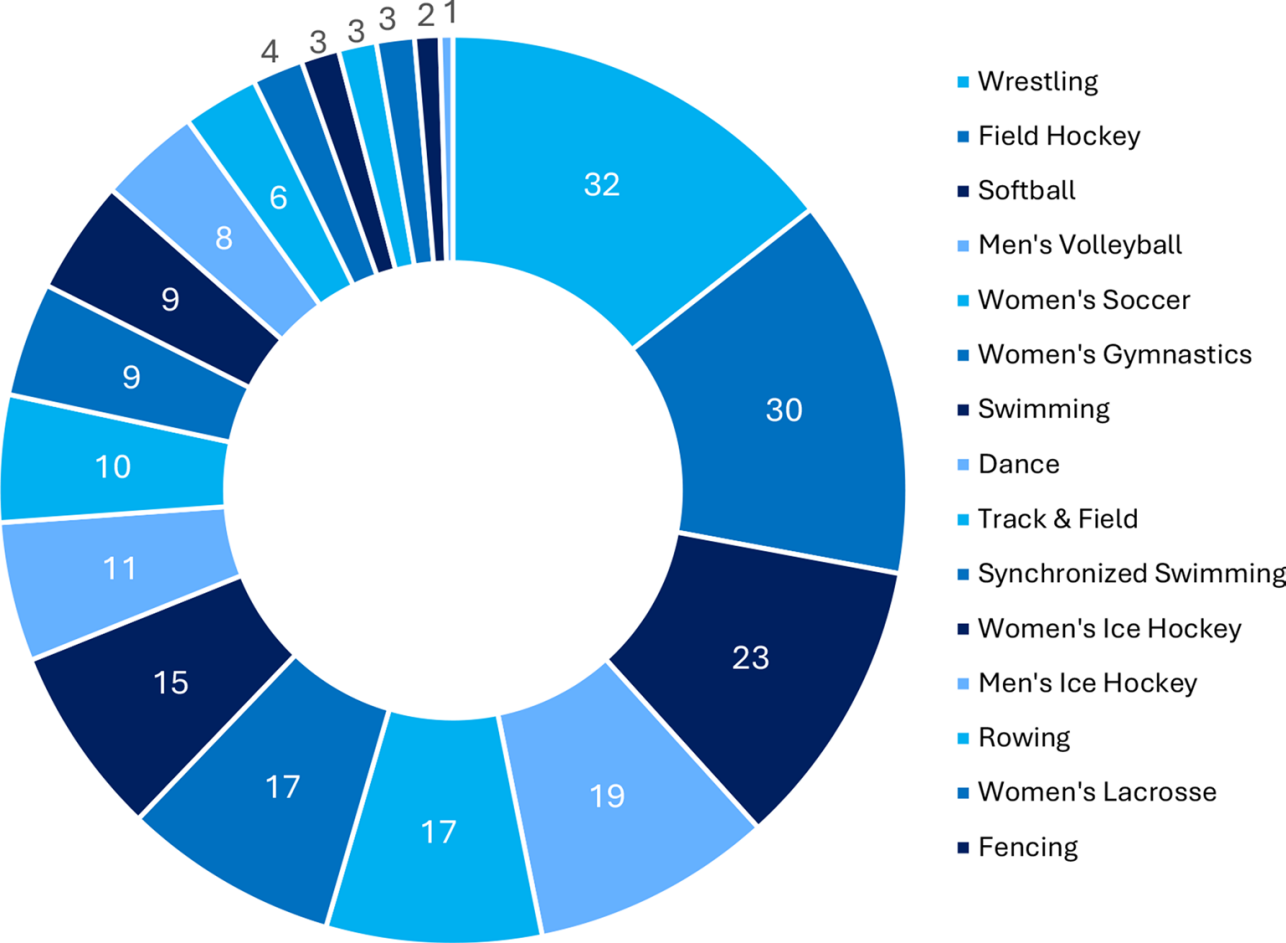
Composition of sports in the sample. Sample size for each sport that was included in data collection and analysis

A total of 247 survey responses were received. Incomplete responses were excluded from all analyses (*n* = 27). A small number of participants (*n* = 8) were included in ED risk analysis but excluded from LEA risk analysis due to an incomplete response to the LEA screening questions. Therefore, the complete sample size for ED risk screening was 220 athletes (76 males, 144 females) across 19 sports, and the complete sample size for LEA risk screening was 212 athletes (72 males, 140 females) across 19 sports. Participant demographics are reported in Table 2. The specific sport breakdown in the sample is presented in Fig. 2.

**Table 2:** Participant demographics

	*N* (%)	Mean Age (years)
Total	220 (100)	19.89 ± 1.42
Males	76 (34.5)	20.04 ± 1.53
Females	144 (64.5)	19.81 ± 1.36

Sample size demographic characteristics

Overall, 38.18% of the athletes in the sample screened positive for ED risk (Table 3). More females screened positive on the EDSA (*p* = 0.002) than males based on chi-squared testing (Table 4). The average score on the EDSA across the sample was notably close to the positive screen score of 3.33 (2.92±0.94). Female athletes had a higher average EDSA score (*p* < 0.001) than male athletes.

**Table 3:** Positive screens and average scores for EDSA and LEA by sex, sport type, and sport

	*N* (M/F)	Average EDSA score	Positive EDSA screens % (*n*)	Average LEAF-Q score	Positive LEAF-Q screens % (*n*)	Average LEAM-Q score	Average LEAM-Q sex drive score	Positive LEAM-Q screens % (*n*)	Positive on both EDSA and LEA screens % (*n*)
Total	220	2.92 ± 0.94	38.18 (84)	--	--	--	--	--	22.73 (50)
Male Athletes	76	2.50 ± 0.93	22.37 (17)	--	--	32.86 ± 14.05	1.68 ± 1.81	16.67 (12)	4.17 (3)
Female Athletes	144	3.15 ± 0.87	46.53 (67)	8.80 ± 3.96	62.86 (88)	--	--	--	32.87 (47)
Aesthetic Sports	39 (2/37)	3.30 ± 0.94	59.00 (23)	8.61 ± 4.07	55.60 (20)	14.00	0	0 (0)	40.54 (15)
Endurance Sports	25 (8/17)	2.81 ± 0.95	36.00 (9)	9.44 ± 4.07	75.00 (12)	49.50 ± 18.25	1.33 ± 1.51	16.67 (1)	27.27 (6)
Ball Sports	112 (27/85)	2.88 ± 0.92	33.04 (37)	8.78 ± 3.86	63.86 (53)	33.04 ± 12.34	1.92 ± 1.81	19.23 (5)	24.77 (27)
Wrestling	32 (32/0)	2.86 ± 1.02	40.63 (13)	--	--	30.34 ± 13.19	1.69 ± 2.01	18.75 (6)	3.13 (1)
Men’s Volleyball	19 (19/0)	2.37 ± 0.82	10.53 (2)	--	--	35.67 ± 10.09	2.11 ± 1.91	22.22 (4)	5.56 (1)
Women’s Gymnastics	17 (0/17)	3.35 ± 0.74	64.71 (11)	9.88 ± 3.41	76.47 (13)	--	--	--	52.94 (9)
Women’s Soccer	17 (0/17)	2.83 ± 0.86	23.53 (4)	8.47 ± 3.10	52.94 (9)	--	--	--	17.65 (3)
Dance Team	11 (0/11)	3.73 ± 0.63	81.82 (9)	7.82 ± 4.47	36.36 (4)	--	--	--	36.36 (4)
Softball	23 (0/23)	3.25 ± 0.89	52.17 (12)	8.91 ± 4.80	68.18 (15)	--	--	--	40.91 (9)
Field Hockey	30 (0/30)	3.26 ± 0.87	50.00 (15)	9.62 ± 3.42	72.41 (21)	--	--	--	41.38 (12)
Swimming	15 (6/9)	2.86 ± 0.86	28.57 (4)	8.89 ± 4.04	66.67 (6)	43.00 ± 25.05	1.60 ± 1.14	0 (0)	14.29 (2)
Other Sports	56 (19/37)	2.59 ± 0.94	25.00 (14)	7.97 ± 4.29	57.14 (20)	31.65 ± 14.78	1.24 ± 1.44	11.76 (2)	17.31 (9)

Presents all outcomes data (prevalence of ED and LEA risk and average scores on screening tools) in the overall sample and divided into each sex, sport type, and sport. EDSA, Eating Disorder Screen for Athletes; ED, eating disorder; LEA, low energy availability; M, males; F, females; LEAF-Q, Low Energy Availability in Females Questionnaire; LEAM-Q, Low Energy Availability in Males Questionnaire

**Table 4:** Statistical significance of average scores and prevalence rates by sex, sport type, and specific sport

	EDSA				LEA			
	Average Score	p	Prevalence	p	Average Score	p	Prevalence	p
Males	2.50 ± 0.93	*p* < 0.001	22.37%	*p* = 0.002	1.68 ± 1.81	N/A	16.67%	*p* < 0.001
Females	3.15 ± 0.87		46.53%		8.80 ± 3.96		62.86%	
Aesthetic	3.30 ± 0.94	*p* = 0.048 (ANOVA) *p* = 0.044 between aesthetic and ball sports	59.00%	*p* = 0.016	8.61 ± 4.07 (LEAF) 0 ± 0 (LEAM)	N/A	54.05%	*p* = 0.871
Endurance	2.81 ± 0.95		36.00%		9.44 ± 4.07 (LEAF) 1.33 ± 1.51 (LEAM)		59.09%	
Ball	2.88 ± 0.92		33.04%		8.78 ± 3.86 (LEAF) 1.92 ± 1.81 (LEAM)		53.21%	
Wrestling	2.86 ± 1.02	*p* < 0.001 (ANOVA) men’s volleyball sig. different from women’s gymnastics (*p* = 0.016), dance (*p* = 0.001), softball (*p* = 0.024), field hockey (*p* = 0.016)	40.63%	*p* = 0.001	1.69 ± 2.01 (LEAM)	*p* = 0.568 (ANOVA) for female sports *p* = 0.145 (t-test) for male sports	18.75%	*p* < 0.001
Men’s Volleyball	2.37 ± 0.82		10.53%		2.11 ± 1.91 (LEAM)		22.22%	
Women’s Gymnastics	3.35 ± 0.74		64.71%		9.88 ± 3.41 (LEAF)		76.47%	
Women’s Soccer	2.83 ± 0.86		23.53%		8.47 ± 3.10 (LEAF)		52.94%	
Dance Team	3.73 ± 0.63		81.82%		7.82 ± 4.47 (LEAF)		36.36%	
Softball	3.25 ± 0.89		52.17%		8.91 ± 4.80 (LEAF)		68.18%	
Field Hockey	3.26 ± 0.87		50.00%		9.62 ± 3.42 (LEAF)		72.41%	

Presents all outcomes data (prevalence of ED and LEA risk and average scores on screening tools) that were tested for statistical analysis divided into each sex, sport type, and sport and reports the p values for all data analysis comparisons that were evaluated. EDSA, Eating Disorder Screen for Athletes; LEA, low energy availability; N/A, not applicable; LEAF, Low Energy Availability in Females Questionnaire; LEAM, Low Energy Availability in Males Questionnaire; ED, eating disorder. *p* < 0.05 was set as the level of significance. Statistical testing of LEA average scores was not conducted by sex or sport type since males and females were evaluated for LEA on different screening tools and therefore received different scores on different scales for this measure. Male average LEA scores are the average score on the sex drive subsection of the LEAM-Q for the specified group

**Fig. 3:**
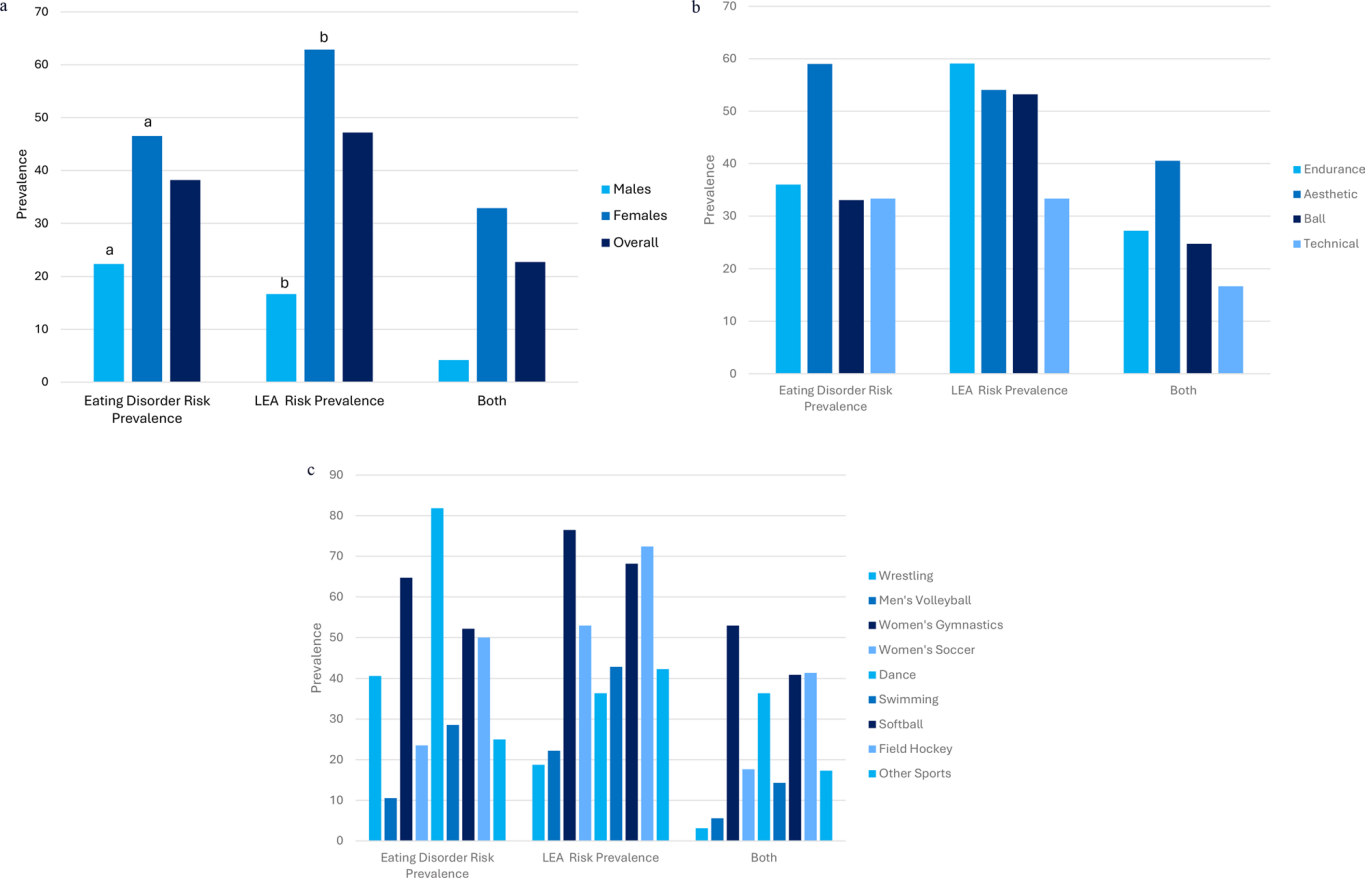
(**a**): Prevalence of ED Risk and LEA Risk by Sex. (**b**): Prevalence of ED Risk and LEA Risk by Sport Type (**c**): Prevalence of ED Risk and LEA Risk by Specific Sport. Graphical presentation of the prevalence of ED risk, LEA risk, and risk of both conditions by sex, sport type, and specific sport. ED, eating disorder; LEA, low energy availability. (**a**) Prevalence rates with the same letters are significantly different (a: *p* = 0.002; b: *p* < 0.001); ((**b**) The prevalence rate of ED risk by sport type is significantly different (*p* = 0.016); ((**c**) The prevalence rate of ED risk and LEA risk by sport is significantly different (ED risk: *p* = 0.001; LEA risk: *p* < 0.001)

Out of all athletes, 47.17% screened positive for LEA risk. Significantly more female athletes screened positive for LEA then male athletes (*p* < 0.001). Notably, the average score on the LEAF-Q was above the LEAF-Q positive screen cut-off score of 8.00 (8.80±3.96), and almost a third of female athletes screened positive for both ED risk and LEA risk. The prevalence rates for ED risk and LEA risk by sex, sport type, and specific sport are compared in Fig. 3.

Aesthetic sport athletes had the highest proportion of ED risk and highest average EDSA score by sport type. Sport types differed significantly in ED positive screens (*p* = 0.016) and average EDSA score (*p* = 0.038), with aesthetic sport athletes having a significantly higher average EDSA than ball sport athletes based on Tukey’s post-hoc testing (*p* = 0.044). A Welch’s test confirmed the finding (*p* = 0.048).

The distribution of positive screens for LEA was not statically significant across sport types (*p* = 0.891). Endurance sport athletes had the highest prevalence of LEA risk, although LEA prevalence rates were above 50% for all 3 sport types. Female endurance sport athletes had the highest prevalence of LEA risk with 75.00% screening positive on the LEAF-Q while male ball sport athletes had the highest prevalence of LEA risk with 19.23% screening positive on the LEAM-Q.

The average score on the LEAF-Q was higher than the at-risk cut-off score of 8.00 for all sport types, with female endurance sport athletes scoring the highest on the LEAF-Q, on average, followed by female ball sport and female aesthetic sport athletes. Male ball sports athletes had the highest average score on the sex drive subsection of the LEAM-Q compared to male endurance sport and male aesthetic sport athletes.

Aesthetic sport athletes had the highest proportion of positive screens on both questionnaires at 40.54% while about a quarter of endurance sport and ball sport athletes screened positive on both questionnaires.

Of all sports with data for at least 10 athletes, dance team athletes had the highest proportion of positive screens on the EDSA, followed by women’s gymnastics, with men’s volleyball having the lowest proportion of positive screens. The distribution of positive screens for EDSA was significantly different across specific sports (*p* = 0.001).

Four sports had an average EDSA score above or within one tenth of the 3.33 cut-off score for a positive screen. The dance team had the highest average EDSA score by specific sport, while men’s volleyball had the lowest. The average score for EDSA was significantly different across specific sports (*p* < 0.001) based on ANOVA analysis. Tukey’s post-hoc testing revealed that the average EDSA score for men’s volleyball was significantly lower than the average EDSA scores for dance team (*p* = 0.001), field hockey (*p* = 0.011), softball (*p* = 0.024), and women’s gymnastics (*p* = 0.016). A test of homogeneity of variances showed that there were equal variances across groups, so a Welch’s test was not needed.

Of all specific sports with data for at least 10 athletes, women’s gymnastics had the highest proportion of athletes screen positive for LEA risk while wrestling had the lowest proportion. The distribution of positive screens for LEA was significantly different across specific sports (*p* < 0.001). A higher proportion of athletes screened positive for LEA risk than ED risk in all sports aside from dance team and wrestling athletes.

Over half of the surveyed women’s gymnastics athletes screened positive on both screening tools while wrestling had the lowest proportion of athletes screen positive for both conditions. Of note, all the dance team athletes who screened positive for LEA risk also screened positive for ED risk.

On average, all women’s sports teams surveyed scored above the 8.00 cut-off score for a positive screen on the LEAF-Q other than dance, which scored within 0.2 of the cut-off score. Women’s gymnastics had the highest average score on the LEAF-Q. However, the difference in average scores on the LEAF-Q was not statistically significant across specific sports (*p* = 0.568). Men’s volleyball had the highest average sex drive subsection score on the LEAM-Q compared to wrestling and swimming. Average scores on the sex drive subsection of the LEAM-Q were not significantly different between men’s volleyball and wrestling (*p* = 0.145). Note that swimming was not included in significance testing for either sex because the sample for this sport was split between the two sexes.

A sport type first identified for evaluation was technical sports; however, the response rate for athletes in this sport type was too low for analysis, as the group included only 6 athletes (3 female golfers, 1 female fencer, and 2 male fencers). Out of these athletes, 33.33% screened positive for ED risk, 33.33% for LEA risk, and 16.67% for both conditions. The average EDSA score for this sport type was 2.86 ± 0.76 with an average LEAF-Q score of 6.25 ± 3.30 and average LEAM-Q sex drive score of 1.50 ± 0.71.

The other sports category was not included in statistical analysis due to its composition of athletes from various sports. However, it is important to note that a quarter of these athletes screened positive on the EDSA, and over 50% of female athletes in this category screened positive for LEA risk. The average score on the LEAF-Q for female athletes was just below the cut-off score for a positive screen (7.97 ± 4.29). Of the athletes in the other sports group, 17.31% screened positive for both ED and LEA risk.

## Discussion

This study aimed to determine the prevalence rates of eating disorder risk and low energy availability risk in collegiate athletes at a large Division 1 university in the Midwest. The prevalence rates were further evaluated by sport, sport type, and sex to evaluate how risk differs amongst these variables. This information could help build more targeted and personalized approaches to identify and improve eating behaviors and attitudes in college athletes.

Based on this study’s results, a significant number of collegiate athletes, from both sexes and all identified sports and sport types, were identified as at risk for an ED and/or LEA. More specifically, female athletes were found to be at a greater risk for ED and LEA when compared to male athletes. Aesthetic sport athletes were at the highest risk for an ED while athletes of all sport types were at high risk for LEA. In accordance with aesthetic sport athletes as a sport type having the highest ED risk, dance team and women’s gymnastics had the highest risk for ED out of the sports evaluated in this study. On the other hand, dance team was at the lowest risk for LEA, while women’s gymnastics and field hockey had the highest risk for LEA out of the female sports. For male sports, wrestling had a higher risk for an ED while men’s volleyball had a higher risk for LEA.

Studies conducted by Sundgot-Borgen and Torsviet [4], Thiemann et al. [10], and DiPasquale and Petrie [26] reported ED risk prevalence rates of 8–15%, although the prevalence of ED risk found in this study (38.18%) adds a higher prevalence rate to the literature. Previous research has determined that female athletes tend to be at a greater risk for an ED [3]. The present study adds to this consensus in the literature, as over twice as many female athletes screened positive for ED risk. However, this comparison does not discount the finding that almost a quarter of male athletes surveyed did screen positive for ED risk. The validation study of the EDSA reported average scores of 3.29 and 2.86 in female and male athletes in their sample [16], which is similar to the average scores on the EDSA in the present study with female athletes in the present study also averaging close to the positive screen cut-off score of 3.33.

Most LEA risk prevalence studies report rates of over 50% in female athletes [20–22], to which the prevalence rate of 62.86% found in this study aligns. Torres-McGhee et al. [20] and Sharps et al. [21] found a higher prevalence of LEA risk than ED risk in female athletes, a pattern to which the current study adds evidence. Limited LEA research exists in male athletes, although one study that has previously been conducted in male athletes found an LEA prevalence of 16.67% (^23^). This study adds to the evidence that male athletes do suffer from LEA, though potentially at a higher rate than previously reported. Currently, little research exists comparing the prevalence of LEA risk between male and female athletes within the same population, a novel aspect of the current study. While females had a significantly higher prevalence of LEA risk than males, this research provides evidence that LEA still affects male athletes and, in turn, male athletes can develop REDs from a prolonged energy imbalance.

Other studies have reported that aesthetic sport athletes have a significantly higher prevalence of ED risk and diagnosis than other sport types [4, 10, 11, 15, 27]. It is proposed that emphasis on leanness, revealing sport uniforms, early age of sport specialization, and the role appearance plays on judgment of performance are all sport-specific risk factors for eating disorders in aesthetic sports [10, 28]. While individual pairings of sport types were not assessed for significant differences, ED risk prevalence was significantly different by sport type, and the prevalence of ED risk in aesthetic sport athletes was greater than the prevalence in the other two sport types by over 20%.

LEA risk did not significantly differ by sport type. Over half of the athletes in all three sport types were at risk for LEA, and the average LEAF-Q scores for aesthetic, endurance, and ball sport athletes were all above the positive cut-off score of 8, with endurance sport athletes having the highest average LEAF-Q score (9.44 ± 4.07). The validation study of the LEAF-Q found a 62.22% prevalence of LEA risk in endurance athletes—which closely matches the result in this study (59.09%)—but no research has evaluated LEA risk in female athletes from other sport types or in male athletes by sport type [22].

Previous studies have also identified a high prevalence of ED risk in dance team and women’s gymnastics while findings are mixed amongst ball sport athletes [17, 20, 29]. Similarly, the current study adds evidence to a high prevalence of ED risk in dance team and women’s gymnastics athletes as these two sports had the two highest ED risk prevalence rates by sport, while some ball sports in the present study had high prevalence of ED risk (50% or greater) and other ball sports had a lower prevalence of ED risk (less than 25%). In contrast, little research exists evaluating ED risk by sport in male athletes and none exists regarding LEA risk by sport in male athletes. A previous meta-analysis found disordered eating to be a prevalent issue in male wrestlers, a conclusion to which the present study can add as almost half of the surveyed wrestlers screened positive for ED risk [30].

Most subgroups identified in this study had a higher proportion of athletes at risk for LEA than for an ED with male, aesthetic sport, dance team, and wrestling athletes the only subgroups that had a higher prevalence of ED risk than LEA risk. Evaluation of LEA in male athletes is still in its infancy, so it is unknown whether male athletes truly had a lower prevalence of LEA risk, or if the measurement of LEA risk in male athletes is less robust than the measurement of ED risk. The two risk proportions were similar in aesthetic sport athletes, and 40.54% of these athletes screened positive on both questionnaires (the highest proportion of any sport type), pointing to a potential association between the two conditions in this group. Overall, higher proportions of LEA risk in most sports could indicate that athletes across many sports have high training demands that could lead to an energy imbalance with or without an ED. While the other sports category was not analyzed due to its heterogeneity, it is important to note that athletes from a variety of sports can be at risk for an ED and LEA.

Several of the limitations in this study were related to the screening tools used in data collection. Screening tools are intended for use in larger populations to identify individuals for closer monitoring and follow up, but errors are inherently part of the process. The LEAM-Q, while validated, is a relatively new screening tool with limited use, and factors such as length, question content, and scoring criteria may have affected athlete answers and screening outcomes [5, 23, 25]. The current conceptual models for LEA and REDs focus on sex hormones and reproductive system responses to LEA [5, 22, 23]. However, other systems are affected by LEA. Typical LEA signs and symptoms often have other possible etiologies—such as polycystic ovary syndrome (PCOS) leading to menstrual dysfunction in women or exercise hypogonadal male condition (EHMC) leading to low testosterone in male athletes—which can often not be evaluated from screening tools alone [5, 22, 23, 31]. Additionally, athletes were routed to an LEA questionnaire based on their sex assigned at birth response, but situations in which an athlete may not fall into the typical gender and sex assigned at birth societal norms present further evidence to the limitations of the LEA screening tools and the sex hormone focus linked to LEA [5, 22, 23, 25]. Other limitations to data analysis include the number of athletes who answered per team—which affected the proportions of positive and negative screens—and the time of year in which the screening was administered—which affected the training demands an athlete currently faced.

There are several opportunities for further research related to both the screening tools themselves and practical application of these findings within athletic departments. For example, future studies should expand the sports included to capture this data across a breadth of athletic teams. Data can be collected for all teams during the same season of training or collected throughout the year to assess any data trends with changes in training seasons. Researchers should consider administering screening measures for these conditions during a dedicated time, such as with yearly physicals or scheduled medical assessments, to improve compliance and reduce needed time outside of these dedicated and medically focused events. Different ED and LEA screening tools can be used within the same population, and results of risk prevalence can be compared among the various screening tools. Additionally, screening results can be compared to physiological markers of LEA, such as bone density measured by dual x-ray absorptiometry (DXA). Researchers should also explore standardized implementation of screening protocols that are both assessable and manageable in collegiate settings as an intervention to evaluate how protocols may affect detection of ED and LEA in athletes.

Screening for ED and LEA can help sports dietitians and other members of the performance paradigm detect athletes at risk for these conditions to receive further evaluation and, if needed, treatment to improve overall health and athletic performance. It is important that athletes and athlete support staff are aware of the signs, symptoms, and impact of ED and LEA. Improved education and scalable detection methods can be used by healthcare professionals and athletic support staff to increase early recognition and treatment of ED and LEA, leading to improved outcomes [3, 5, 6, 24].

## Conclusions

The results of this study add to the existing evidence that many collegiate athletes are at risk for eating disorders and low energy availability. Based on the results of this study, female athletes are at a greater risk for ED and LEA than male athletes. Athletes from all sport types are at high risk for LEA, while aesthetic sport athletes are at the highest risk for an ED. Dance team and women’s gymnastics had the highest ED risk and LEA risk, respectively.

Both ED and LEA have significant effects on overall health and athletic performance, making early detection and treatment crucial [3, 5]. More research into the prevalence rates of these conditions in athletes is needed. Additionally, the implementation of standardized screening protocols and the evaluation of the efficacy of these protocols are necessary to detect and treat athletes with eating disorders and low energy availability to protect their overall health and athletic performance.

## Data Availability

No datasets were generated or analysed during the current study.

## Declarations

### Author information

LP: MDN, RDN, LD, CPT; Performance Nutrition Fellow, University of Delaware Sport Performance, Newark, DE, USA. JK: PhD, RDN, LD; Associate Professor, Department of Human Sciences, The Ohio State University, Columbus, OH, USA. CS: PhD, RDN, CSCS; Assistant Professor, Department of Human Sciences, The Ohio State University, Columbus, OH, USA

## Abbreviations

ANOVA: Analysis of variance
DXA: Dual x-ray absorptiometry
ED: Eating disorder(s)
EDSA: Eating Disorder Screen for Athletes
EHMC: Exercise hypogonadal male condition
IOC REDs CAT2: International Olympic Committee Relative Energy Deficiency in Sport Clinical Assessment Tool 2
LEA: Low energy availability
LEAF-Q: Low energy availability in females questionnaire
LEAM-Q: Low energy availability in males questionnaire
NCAA: National Collegiate Athletic Association
PCOS: Polycystic ovary syndrome
REDs: Relative Energy Deficiency in Sport
